# Dialectical behavioral therapy-based group treatment versus treatment as usual for adults with attention-deficit hyperactivity disorder: a multicenter randomized controlled trial

**DOI:** 10.1186/s12888-022-04356-6

**Published:** 2022-11-28

**Authors:** Anne Halmøy, Anna Edith Ring, Rolf Gjestad, Merete Møller, Bente Ubostad, Tage Lien, Ellen Kathrine Munkhaugen, Mats Fredriksen

**Affiliations:** 1 Division of Psychiatry, Kronstad District Psychiatric Center, Haukeland University Hospital, 5021 Bergen, Norway; 2 Department of Clinical Medicine, University of Bergen, 5020 Bergen, Norway; 3 Division of Psychiatry, Research Department, Haukeland University Hospital, 5021 Bergen, Norway; 4 Center for Research and Education in Forensic Psychiatry, Haukeland University Hospital, 5021 Bergen, Norway; 5 District Psychiatric Center, Østfold Hospital Trust, 1714 Grålum (Sarpsborg), Norway; 6 Regional Resource Center for Autism, AD/HD, and Tourettes Syndrome, Western Norway Regional Health Authority, Haukeland University Hospital, 5021 Bergen, Norway; 7 Division of Mental Health & Addiction, Vestfold Hospital Trust, 3101 Tønsberg, Norway; 8 Regional Resource Center for Autism, AD/HD, and Tourettes Syndrome, South-Eastern Norway Regional Health Authority, Oslo University Hospital, 0424 Oslo, Norway; 9 Norwegian National Advisory Unit On Mental Health in Intellectual Disabilities, Oslo University Hospital, 0424 Oslo, Norway

**Keywords:** Attention-deficit, Hyperactivity disorder (ADHD), Adults, Non-pharmacological treatment, Group therapy, Dialectical behavioral therapy (DBT)

## Abstract

**Background:**

Studies on structured skills training groups have indicated beneficial, although still inconclusive, effects on core symptoms of ADHD in adults. This trial examined effects of Dialectical Behavioral Therapy-based group treatment (DBT-bGT) on the broader and clinically relevant executive functioning and emotional regulation in adults with ADHD.

**Methods:**

In a multicenter randomized controlled trial, adult patients with ADHD were randomly assigned to receive either weekly DBT-bGT or treatment as usual (TAU) during 14 weeks. Subsequently, participants receiving TAU were offered DBT-bGT. All were reassessed six months after ended DBT-bGT. Primary outcomes were the Behavior Rating Inventory of Executive Function (BRIEF-A) and the Difficulties in Emotion Regulation Scale (DERS). Secondary outcomes included self-reported ADHD-symptoms, depressive and anxiety symptoms, and quality of life. We used independent samples t- tests to compare the mean difference of change from pre- to post-treatment between the two treatment groups, and univariate linear models adjusting for differences between sites.

**Results:**

In total, 121 participants (68 females), mean age 37 years, from seven outpatient clinics were included, of whom 104 (86%) completed the 14-week trial. Entering the study, 63% used medication for ADHD. Compared to TAU (*n* = 54), patients initially completing DBT-bGT (*n* = 50) had a significantly larger mean reduction on the BRIEF-A (-12.8 versus -0.37, *P* = 0.005, effect size 0.64), and all secondary outcomes, except for symptoms of anxiety. All significant improvements persisted at 6 months follow-up. Change on DERS did not differ significantly between the groups after 14 weeks, but scores continued to decrease between end of group-treatment and follow-up.

**Conclusions:**

This DBT-bGT was superior to TAU in reducing executive dysfunction, core symptoms of ADHD and in improving quality of life in adults with ADHD. Improvements sustained six months after ended treatment. The feasibility and results of this study provide evidence for this group treatment as a suitable non-pharmacological treatment option for adults with ADHD in ordinary clinical settings.

**Trial registrations:**

The study was pre-registered in the ISRCTN registry (identification number ISRCTN30469893, date February 19^th^ 2016) and at the ClinicalTrials.gov (ID: NCT02685254, date February 18^th^ 2016).

## Background

Attention-Deficit/Hyperactivity Disorder (ADHD) is a common, life-spanning, neurodevelopmental disorder [1] with prevalence estimates around 3% in adults [2]. Individual, health care and societal costs due to consequences of ADHD in adults are significant [3, 4]. Multimodal treatment is preferred for ADHD, both for children and adults [5]. Pharmacological treatment is shown to be effective in reducing core symptoms of ADHD [6] and is recommended as a first-line treatment [7]. However, adults with ADHD often have symptoms and challenges beyond the core symptoms of attention deficits, hyperactivity and impulsivity. Common adjuvant and secondary symptoms among adults with ADHD include lack of organizational skills and coping strategies, difficulties with time management, low self-esteem as a consequence of continuous failure and misunderstandings, problems with emotional regulation, and comorbidity or symptoms from other psychiatric disorders [4, 8–10]. Such problems may be less responsive to medication, and benefits of pharmacological treatment on long-term outcomes may be lower when initiated at later ages [11, 12]. Psychotherapeutic interventions for ADHD should target these adjuvant problems [13].

Cognitive behavioral therapy (CBT), both individually [14, 15], in group settings [16], and combined [17] is so far the most documented non-pharmacological treatment for adults with ADHD [18]. However other treatments like metacognitive therapy and mindfulness have shown promising results [18, 19]. Psychoeducation often forms part of non-pharmacological treatment programs, and may alleviate symptoms in itself [20, 21]. Dialectical behavioral therapy (DBT) includes these aspects, but focuses in addition on acceptance of problems; the term dialectical referring to a balance between acceptance and change of behavior. DBT was originally developed for the treatment of borderline personality disorder (BPD) [22], but adaptations to other disorders have been made, including ADHD [23]. Common traits and symptoms between BPD and ADHD (i.e., impulsivity, emotional instability, and disorganized behavior) make DBT an interesting approach for ADHD. In 2002–04, Hesslinger and colleagues developed a DBT-based group treatment program adapted to adults with ADHD in Germany [24, 25]. This group program differs from the original DBT for BPD by shorter duration (12–14 weeks instead of 1 (-2) years), lack of individual sessions, and more specific focus on ADHD in the psychoeducation and skills training. Their first pilot study (8 participants) [25], and subsequent open, multicentre study (*n* = 72 patients) [26] as well as a later open feasibility study from Sweden (*n* = 98) [27] all showed reductions of both ADHD-symptoms and comorbid symptoms of depression in adults with ADHD after this group treatment. A smaller randomized controlled trial from the Swedish group (*n* = 51) showed that this specific group treatment was more effective in reducing core symptoms of ADHD compared to a loosely structured discussion group, but found no significant difference on comorbid depressive symptoms [28]. The largest study so far of this DBT-treatment, including 433 patients, used a four-armed design to compare the group treatment to general clinical management, combined with medication (methylphenidate) or placebo, respectively, in adults with ADHD [29, 30]. Medication was found more effective in reducing core symptoms of ADHD during the trial however, follow-up studies indicated that the DBT-based group treatment had a more long-lasting effect on general clinical status and quality of life [28, 31]. It can be argued that traditional checklists of core symptoms are more suitable for assessment in trials of medication than of psychotherapy, where the goal is rather on coping strategies than symptom reductions in itself [32]. Furthermore, in DBT-based treatment, two of the main tools, e.g., mindfulness and behavioral analyses, specifically target emotional regulation (ER) and executive functioning (EF), which have shown to be important and independent mediators of impairments in adults with ADHD [33–35]. A pilot study of another DBT-based group treatment of 8 weeks found a positive effect on self-reported EF in college students (*n* = 33) with ADHD [36]. However, none of the larger, clinical studies of DBT-based treatment for ADHD published so far has specifically examined ER or EF. A main motivation for conducting this study was to increase the availability of evidence-based non-pharmacological treatment options for adults with ADHD. Implementation of the group treatment in a general clinical setting was therefore an important aspect of the study design.

The specific objectives of this study were to examine the efficacy of a manualized DBT-based group treatment compared to treatment delivered as usual for adults with ADHD. Our primary hypotheses were that the group treatment would be superior to treatment as usual on self-reported executive functioning and emotional regulation, and secondly, that the group treatment, relative to treatment as usual, would have a larger effect on core symptoms of ADHD, symptoms of depression and anxiety, and quality of life.

## Methods/design

### Study design and participants

The present study is a multicenter parallel group randomized controlled trial (RCT) comparing the effects of a DBT-based group therapy (DBT-bGT) with 'Treatment as usual’ (TAU) for adults with ADHD. Included participants were randomly allocated (ratio 1:1) to either the active DBT-bGT or the control condition TAU by a blinded lottery procedure performed and supervised at each site. After this initial controlled trial, participants in the control group, i.e. who initially received TAU, subsequently underwent the DBT-based group treatment, in an uncontrolled extension phase of the study. For the control group, the post-RCT assessment was thus used as pre-assessment before starting the group treatment, as long as there was less than 2 months between end of the RCT-trial and start of group treatment. Due to summer holiday, some sites did not start the group treatment for the control group within the first 2 months, and in that case, the control group went through a new pre-assessment before starting the group treatment. All participants were then re-assessed 6 months after having received their DBT-bGT.

The study protocol was approved by the Regional Committees for Medical Research (REC South East Norway, ID 2015/01523), and conducted in accordance with ethical standards following the principals of the Declaration of Helsinki. All included participants gave their written informed consent before entering the study.

Estimating sample size based on literature review and a power calculation assuming at least difference of 10% between means of the two independent groups (and SD 15%), gave a need of about 50 participants in each group (alpha = 0.05, power = 0.9). Seven psychiatric adult outpatient clinics in South-Eastern and Western Norway contributed. Clinicians at each site included 16–18 patients between February 20^th^ and December 31^th^ 2016, who were then randomly allocated to either the active DBT-bGT (one group at each site) or the control condition TAU. Inclusion criteria were a clinical diagnosis of ADHD (according to the Diagnostic and Statistical Manual of Mental Disorders-IV), and a minimum age of 18 years. Diagnostic assessment was part of standard diagnostic procedures at the participating clinics, which include confirmatory assessment by a specialist in psychiatry or psychology. Exclusion criteria were ongoing psychiatric disorders and/or psychosocial factors considered to clearly interfere with the patients’ motivation or ability to participate in the group therapy, i.e., ongoing substance or alcohol abuse, psychotic disorder, major depressive or manic episode, and suicidal behavior; organic brain damage, neurological diseases causing mental handicap, intellectual disability (IQ ≤ 70), and pervasive developmental disorder. Information about both ADHD and comorbid conditions was based on a questionnaire to the referring clinician, designed for this study. Participants did not undergo specific diagnostic assessment for this study in particular. However, clinical guidelines and standard clinical practice for diagnostic evaluation in psychiatric outpatient clinics in Norway include the use of diagnostic instruments corresponding to DSM-/ICD-criteria, i.e. the MINI/MINIplus interview for axis-1 psychiatric disorders, SCID-II/5 for personality disorders and DIVA for ADHD.

Patients were allowed to receive pharmacological treatment but should be stabilized on an adequate type of medication and dosage at least 6 weeks before inclusion, and as far as possible avoid changes in medication during the study-period. However, as we also aimed for a naturalistic setting, we did not exclude patients that underwent medication change during the trial, if this was judged as necessary or clinically important by the treating clinician. Instead, we included a question about this in the questionnaire to the referring/treating clinician.

### Intervention

The DBT-bGT was based on a Swedish version of the manual [37] originally developed by Hesslinger et al. [25]. The treatment uses elements from DBT such as psychoeducation, acceptance, mindfulness, and functional behavioral analysis, targeting symptoms and functional problems common in ADHD. It consists of 14 weekly group sessions, each lasting two hours separated by a 15-min break. Each group included 7–9 adult patients with ADHD and two therapists. Group sessions followed a structure with manualized instruction for the therapists and workbook for the patients. A typical session starts by introducing a new mindfulness exercise performed together in the group. The first part of the session then focuses on feedback on last week’s homework of skill training, while the second part introduces a new topic and related homework for the next week. The topics for the different sessions include psychoeducation, mindfulness, functional behavioral analyses, and how to understand and manage different symptoms and aspects of ADHD, e.g. impulsivity, addiction, emotional regulation, self-esteem, and relation to others [28]. Interaction between the participants is important, and the therapists should encourage and balance their feedback and discussion during the session. After each group session, patients received 15–20 min of individual coaching with one of the therapists. This was an add-on, according to a Swedish adaptation of the program [27]. The coaching focuses on adherence to homework related to each participant’s situation and pre-defined goals.

The therapists were health service professionals with various backgrounds: medical doctors/psychiatrists, psychologists, nurses, and some special educators. There were no requirements of former DBT-training, but all therapists had clinical experience and interest in adults with ADHD and/or CBT and /or group treatment. All group therapists participated at a 2-days’ seminar for an introduction to the principles of DBT and the use of the manual, led by one of the main contributors to the Swedish manual and studies on this method. To assure a common understanding and quality of the treatment, the therapists also participated at a minimum of two digital meetings led by the project leader to discuss and get feedback on challenges and practical issues encountered during the trial period.

The control condition of the trial (TAU) also lasted for 14 weeks. TAU was not standardized but rather defined as the treatment that the patient would have received if not included in the project. It could thus vary between both individuals and clinics. The most common treatment for this patient group in outpatients in Norway, consistent with national clinical guidelines, consists of individual consultations delivered by a psychiatrist or psychologist, focusing on psychoeducation and general clinical management, often in combination with medication. To obtain more information about the actual treatment received by the control group, referring clinicians were asked to respond to some questions about frequency and focus of the delivered treatment in the time-period of the trial.

## Outcome measures

### Primary outcomes

Participants were assessed one week before treatment (pre-treatment) as baseline, and one week after the 14-week trial (post-treatment), and then again six months after ended DBT-bGT for all the participants (non-controlled follow-up). Primary outcomes were symptoms of executive functioning (EF) measured by the Behavior Rating Inventory of Executive Functioning Adult version (BRIEF-A) and emotion regulation (ER) according to the Difficulties in Emotion Regulation Scale (DERS). The BRIEF- A consists of 75 items about self-reported executive functioning operationalized in different domains of every-day life [38]. The presence of each item is rated on a 3-point Likert scale from 1 (never) to 3 (often). Several subscales may be calculated, but for the purpose of this study we used the sum score (global executive composite score). The DERS is a questionnaire consisting of 36 statements about thoughts, reactions and behavior related to own emotional state [39]. Participants rate how often the statements apply to them, from ‘almost never’ (0–10%) to ‘almost always’ (91–100%). Scores may be calculated for separate subscales and summed to a total score, the latter used in this study.

### Secondary outcomes

Secondary outcomes were core symptoms of ADHD on the Adult ADHD Rating Scale (ASRS, the original 18-item version), symptoms of depression and anxiety (as defined by the Becks Depression Inventory (BDI) and Becks Anxiety Inventory (BAI), respectively), and quality of life measured by the Adult ADHD Quality of Life Scale (AAQoL). The ASRS [40] grades the presence of core symptoms of ADHD for the last 6 months, on a Likert scale from 0 (never) to 4 (very often). (For this study, the time-period for reported symptoms at the post-trial and follow-up assessments was specified to ‘since last evaluation’ or ‘last month’). The AAQoL [41] is a 29-item questionnaire assessing health related and disease specific measures at different domains of quality of life in adults with ADHD. The BDI*,* version II [42] and the BAI [43] are self-report scales for last week’s occurrence of symptoms for depression and anxiety, respectively.

### Other parameters

As baseline characteristics, we recorded educational level, employment status, and clinical subtype of ADHD, diagnosed comorbid mental disorders, and information about medication for ADHD as reported by the patients’ clinicians. Patients also filled in two screening questionnaires for alcohol- and substance-problems; The Alcohol Use Disorder Identification Test (AUDIT) [44] and The Drug Use Disorder Identification Test (DUDIT) [45], respectively.

### Statistical analyses

Changes in mean scores for outcome measures from pre- to post-treatment within the DBT-bGT and TAU groups, respectively, were analyzed with paired sample t-tests. We used independent samples t- tests to compare the mean difference of change from pre- to post-treatment between the two treatment groups To account for the non-independence and nested nature of data due to participants representing different sites, we used univariate linear models, with site/clinic as a fixed factor, and excluding intercept from the model. This model yields an estimate of the group (= intervention) effect after having controlled for the different levels at each site. Since site and therapists represent the same level in this model (each site had only one group with one set of therapists) we performed the analyses only with site as a fixed factor in the model.

For the non-controlled extension part of the study, we used paired sample t-tests to assess change from baseline to 6-months follow-up, and analyses of variance (ANOVA) for repeated measures to assess change in symptom scores from baseline to post- treatment from the RCT and at 6 months follow-up after group treatment for all participants.

All analyses were pre-specified and performed with the software package IBM SPSS Statistics (version 24). Standardized effect sizes (ES) of the treatment were calculated by dividing the mean difference in symptom scores from pre-to post treatment with the pooled standard deviation (SD) of the respective measure, and reported as Cohen’s d. The significance threshold was set at 5% (two-tailed) and we used two-sided 95% confidence intervals (CI). Analyses included, and were restricted to, participants with actual responses on each of the respective questionnaires, i.e., excluding patients with missing values analysis-by-analysis.

## Results

### Sample characteristics

**Fig. 1:**
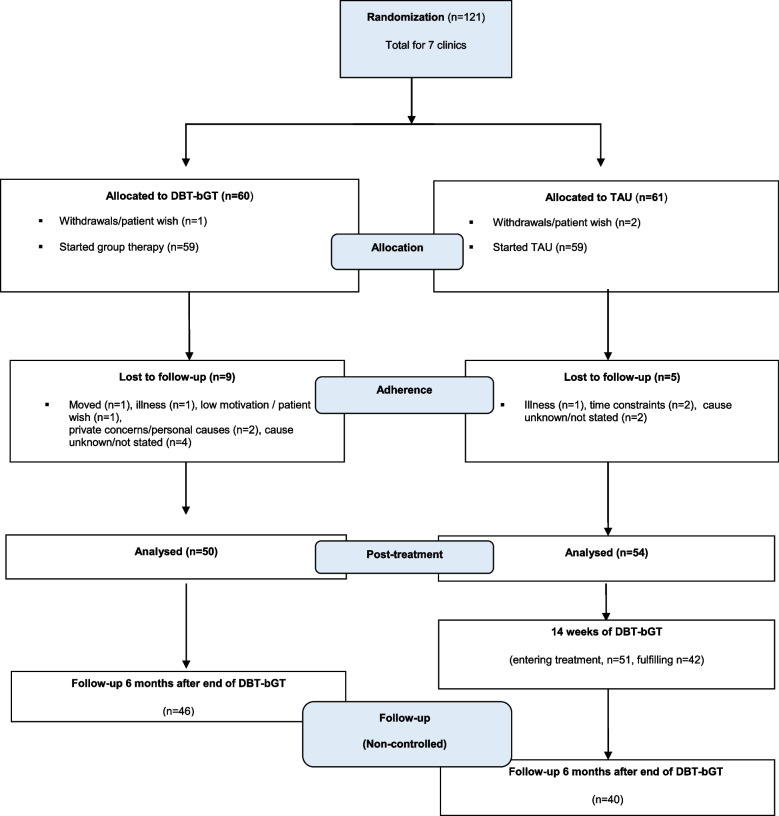
Flowchart of study design and included patients. Patients randomized to either dialectical behavioral therapy based group treatment (DBTb-GT) or Treatment as usual (TAU)

Of the 121 randomized patients, three withdrew before starting the treatment and 104 (86%) completed the 14-week trial (Fig. 1). Mean age was 37 years (range 21–59) and 56% were female. Less than one of five were full time employed or student, and one of three were out of work (unemployed, on sick leave, receiving a disability pension or work assessment allowance). The most frequent subtype of ADHD was the combined (68%), followed by the inattentive (22%). The mean total ASRS score was 46.8 (range 0–72). Most patients (88%) had tried pharmacological treatment for ADHD, and 63% were still using ADHD-medication when entering the study. Two thirds had at least one comorbid psychiatric diagnosis. At baseline, patients allocated to DBTb-GT showed a statistically higher mean score of depressive symptoms (BDI score 20.1 versus 15.1, *p* = 0.02), and AUDIT and DUDIT scores than the TAU group. Other clinical or sociodemographic variables did not differ significantly between the two treatment groups at baseline (Table 1).

**Table 1:** Sociodemographic and clinical characteristics of participants at baseline

	DBTb-GT (*n* = 60)	TAU (*n* = 61)
Mean age, years (min–max)	36.5 (21–59)	37.5 (21–57)
Gender (female/male)	32/28	36/25
Educational level		
University/college, n (%)	10 (16.7)	9 (14.8)
Lower/Other, n (%)	50 (83.3)	52 (85.2)
Main occupation (full-time), n (%)		
In work/studying	7 (11.7)	15 (24.6)
Unemployed/sick leave	4 (6.7)	5 (8.2)
Work assessment allowance/disability pension	17 (28.3)	15 (24.6)
Age (first) diagnosed with ADHD, mean (SD)	32.8 (10.7)	33.2 ( 11.7)
ADHD sub-type, n (%)		
Combined	43 (71.7)	39 (63.9)
Inattentive	11 (18.3)	16 (26.2)
Hyperactive/Impulsive	2 (3.3)	0
Not specified (incl. sub-threshold cases)	2 (3.3)	2 (3.3)
Comorbid psychiatric diagnoses, mean number (SD)	1.46 (1.26)	1.19 (1.20)
Pharmacological treatment for ADHD, n (%)		
Ever tried	53 (88.3)	54 (88.5)
Use at inclusion, yes	38 (63.3)	38 (62.3)
Effect of medication (reported by clinician)		
Very good/good	36 (67.9)	44 (81.5)
No /unsure effect	12 (22.6)	10 (18.5)
Symptom-scores, mean (SD) ^‡^		
BRIEF-A (*n* = 116, 59/57)	151.8 (20.3)	147.5 (23.3)
DERS (*n* = 113, 58/59)	104.4 (25.3)	105.5 (25.2)
ASRS (*n* = 113, 59/54)	46.9 (8.2)	46.7 (9.5)
AAQoL (*n* = 100, 51/49)	49.1 (13.8)	53.0 (14.0)
BDI (*n* = 112, 56/56)	20.1 (10.9)	15.1 (11.2)
BAI (*n* = 112, 56/56)	15.3 (9.5)	12.5 (8.8)
AUDIT (*n* = 115, 58/57)	7.3 (6.2)	5.1 (4.5)
DUDIT (*n* = 116, 58/58)	1.9 (4.9)	0.5 (1.4)

*DBTb-GT* dialectical behavioral therapy-based group treatment, *TAU* treatment as usual, *BRIEF-A* Behavior Rating Inventory of Executive Function – Adult Version, total sum score, *DERS* Difficulties in Emotion Regulation Scale, total sum score, *ASRS* Adult ADHD Self-Report Scale, total sum score, *AAQoL* Adult ADHD Quality of Life Questionnaire, total sum score, *BDI* Beck Depression Inventory, total sum score, *BAI* Beck Anxiety Inventory, total sum score, *AUDIT* Alcohol Use Disorder Identification Test, total sum score, *DUDIT* Drug Use Disorder Identification Test, total sum score, *SD* standard deviation, *n* number of participants

^‡^ number of responders (n total, n group therapy/n TAU) varies between questionnaires, due to missing data for some participants

## Outcomes at end of the 14-week trial

### Primary outcomes

Compared to individuals receiving TAU (*n* = 54), patients completing DBT-bGT (*n* = 50) reported a significantly larger mean improvement of EF (reduction on the BRIEF total score -12.8 versus -3.7, respectively). The difference in change between the groups was statistically significant (*p* < 0.001) with an ES of = 0.64, which according to common interpretations of Cohen’s effect sizes corresponds to a medium effect. The proportion of patients with an actual reduction on the BRIEF total score was 74.0% and 53.8% for the DBTb-GT and TAU, respectively (Pearson chi-square (χ^2^) 4.48, p = 0.034). The proportion of patients with a BRIEF-score in the clinical range (i.e. BRIEF T-score of 65 or more) decreased significantly from 81.4% to 64.0% (χ^2 =^ 6.3, *p* = 0.019) in the DBT-group compared to a slight, boarder-line significant increase from 75.4% to 77.4% (χ^2^ = 5.2, *p* = 0.051) in the TAU-group, from before to after treatment.

Participants of the DBT-group also showed a larger intra-group mean reduction on the DERS total score than the TAU group (-7.5, *p* = 0.03 vs. -3.9, *p* = 0.15, respectively), but the difference in change between the two groups was not statistically significant (*p* = 0.39) (Table 2).

**Table 2:** Outcome measures before and after receiving dialectical behavioral therapy-based group treatment, and treatment as usual

	Pre-treatment	Post-treatment	Change pre-post	Statistics for analyses within group ^a^		Statistics for analyses between groups ^b^	
Outcome measure (n)	Mean (SD)	Mean (SD)	Mean (SD)	t (df)	*p*	*p*	Cohen’s d ^c^
BRIEF						0.002	0.64
Group therapy (50)	152.1 (20.6)	139.3 (25.1)	-12.8 (19.6)	4.6 (49)	< 0.001		
TAU (52)	145.8 (22.5)	145.5 (25.8)	-0.37 (19.2)	0.1 (51)	0.891		
DERS						0.393	0.18
Group therapy (44)	104.0 (26.4)	96.5 (28.4)	-7.5 (21.6)	2.3 (43)	0.026		
TAU (48)	104.4 (26.7)	100.5 (28.1)	-3.9 (18.4)	1.5 (47)	0.147		
ASRS						< 0.001	1.01
Group therapy (49)	46.6 (8.3)	38.7 (9.0)	-7.9 (9.5)	5.8 (48)	< 0.001		
TAU (47)	45.6 (8.9)	45.4 (9.3)	-0.17 (5.1)	0.2 (46)	0.820		
AAQoL						0.004	0.64
Group therapy (44)	48.6 (14.1)	57.5 (16.8)	9.0 (18.0)	-3.3 (43)	0.002		
TAU (45)	53.6 (14.0)	52.2 (17.3)	-1.48 (14.7)	0.7 (44)	0.505		
BDI						0.005	0.58
Group therapy (48)	20.9 (11.2)	14.5 (11.8)	-6.4 (11.1)	4.0 (47)	< 0.001		
TAU (49)	13.9 (10.8)	13.6 (10.4)	-0.35 (9.9)	0.3 (48)	0.807		
BAI						0.169	0.28
Group therapy (48)	15.5 (9.7)	13.4 (11.2)	-2.2 (8.4)	1.8 (47)	0.082		
TAU (50)	11.9 (8.7)	12.4 (10.4)	0.4 (10.1)	-0.3 (49)	0.759		

^a^ Mean difference of sum score from pre- to post-treatment within each group with standard deviation (SD) and t (df) from paired sample

^b^ *p*-value from independent sample t-test of mean difference of change between groups

^c^ Effect size for the difference in change between groups, reported as Cohen’s d. *n* Number of included patients in the paired sample t-test for each outcome measure, *SD* standard deviation, *TAU* treatment as usual, *BRIEF-A* Behavior Rating Inventory of Executive Function – Adult Version, total sum score, *DERS* Difficulties in Emotion Regulation Scale, total sum score, *ASRS* Adult ADHD Self-Report Scale, total sum score, *AAQoL* Adult ADHD Quality of Life Questionnaire, total sum score, *BDI* Beck Depression Inventory, total sum score, *BAI* Beck Anxiety Inventory, total sum score

### Secondary outcomes

Compared to individuals receiving TAU, patients receiving the DBT-bGT reported a significant improvement of core symptoms of ADHD (ASRS total score, -7.9 vs. -0.17, *p* < 0.001, ES = 1.01), depressive symptoms (BDI total, -6.4 vs. 0.35, *p* < 0.001, ES = 0.58), and quality of life (AAQol total, 9.0 vs. -1.48, p = 0.004, ES = 0.63) (Table 2). Conventional interpretations of Cohen’s d thus indicate a large effect (ES > 0.8) on core symptoms of ADHD, and moderate (ES > 0.5) on symptoms of executive functioning and depression. The BAI scores did not change significantly for neither of the groups during the treatment period.

Analyses controlling for clinic-level showed that, despite some variation between clinics, DBTb-GT was still superior to TAU in reducing symptoms on BRIEF (β -12.5, *p* = 0.002), ASRS (β -7.5, *p* < 0.001), AAQoL (β 10.5, *p* = 0.005) and BDI (β -5.9, *p* = 0.008). As for the uncontrolled analyses, the effect on DERS (β -3.5, *p* < 0.406) and BAI (β -2.5, *p* = 0.179) were not statistically significant.

### Follow-up at 6 months

Overall, the observed symptom reductions from pre- to post-treatment for the DBT-group persisted at 6 months follow-up. A continued improvement was found for the BRIEF and DERS scales, where 28% and 39% of the total symptom reduction, respectively, occurred after ended treatment (Table 3). For the BDI and AAQoL there was a slight decline of the observed improvements at post-treatment, but still with a significant improvement relative to baseline (Table 3).

**Table 3:** Symptom change from baseline to follow-up for participants randomized to dialectical behavioral therapy-based group treatment

	Pre-treatment	Post-treatment	Follow-up 6 months after ended group treatment	Change pre-treatment to 6 months follow-up	Change post-treatment to 6 months follow-up	Statistics for the change within group from pre-treatment to 6 months follow-up ^a^		Statistics for the change from post-treatment to 6 months follow-up ^a^	
Outcome measure ^b^	Mean (SD)	Mean (SD)	Mean (SD)	Mean (SD)	Mean (SD)	t (df)	*p*	t (df)	*p*
BRIEF									
Group therapy	152.6 (21.1)	139.3 (25.1)	134.2 (26.3)	-18.4 (21.5)	-5.1 (20.5)	5.8 (45)	< 0.001	1.7 (45)	0.104
DERS									
Group therapy	104.0 (26.4)	96.5 (28.4)	91.7 (26.7)	-12.3 (19.6)	-4.6 (18.6)	4.1 (43)	< 0.001	1.6 (43)	0.113
ASRS									
Group therapy	46.8 (8.5)	38.6 (9.1)	36.5 (11.2)	-10.3 (11.0)	-2.0 (8.9)	6.3 (44)	< 0.001	1.6 (44)	0.128
AAQoL									
Group therapy	48.7 (14.0)	57.8 (16.9)	57.3 (19.2)	8.6 (18.3)	-1.4 (17.6)	-3.0 (39)	0.005	0.5 (42)	0.603
BDI									
Group therapy	20.3 (10.4)	13.0 (10.9)	14.6 (13.2)	-5.8 (11.5)	1.2 (11.6)	3.3 (43)	0.002	-0.7 (45)	0.478
BAI									
Group therapy	15.1 (9.8)	12.8 (11.1)	11.4 (9.8)	-3.8 (7.7)	-1.5 (6.5)	3.2 (43)	0.002	1.6 (44)	0.127

*BRIEF-A* Behavior Rating Inventory of Executive Function – Adult Version, total sum score, *DERS* Difficulties in Emotion Regulation Scale, total sum score, *ASRS* Adult ADHD Self-Report Scale, total sum score, *AAQoL* Adult ADHD Quality of Life Questionnaire, total sum score, *BDI* Beck Depression Inventory, total sum score, *BAI* Beck Anxiety Inventory, total sum score, *SD* Standard deviations

^a^ From paired sample t-test

^b^ Number of included participants for each analysis equals df + 1

**Fig. 2:**
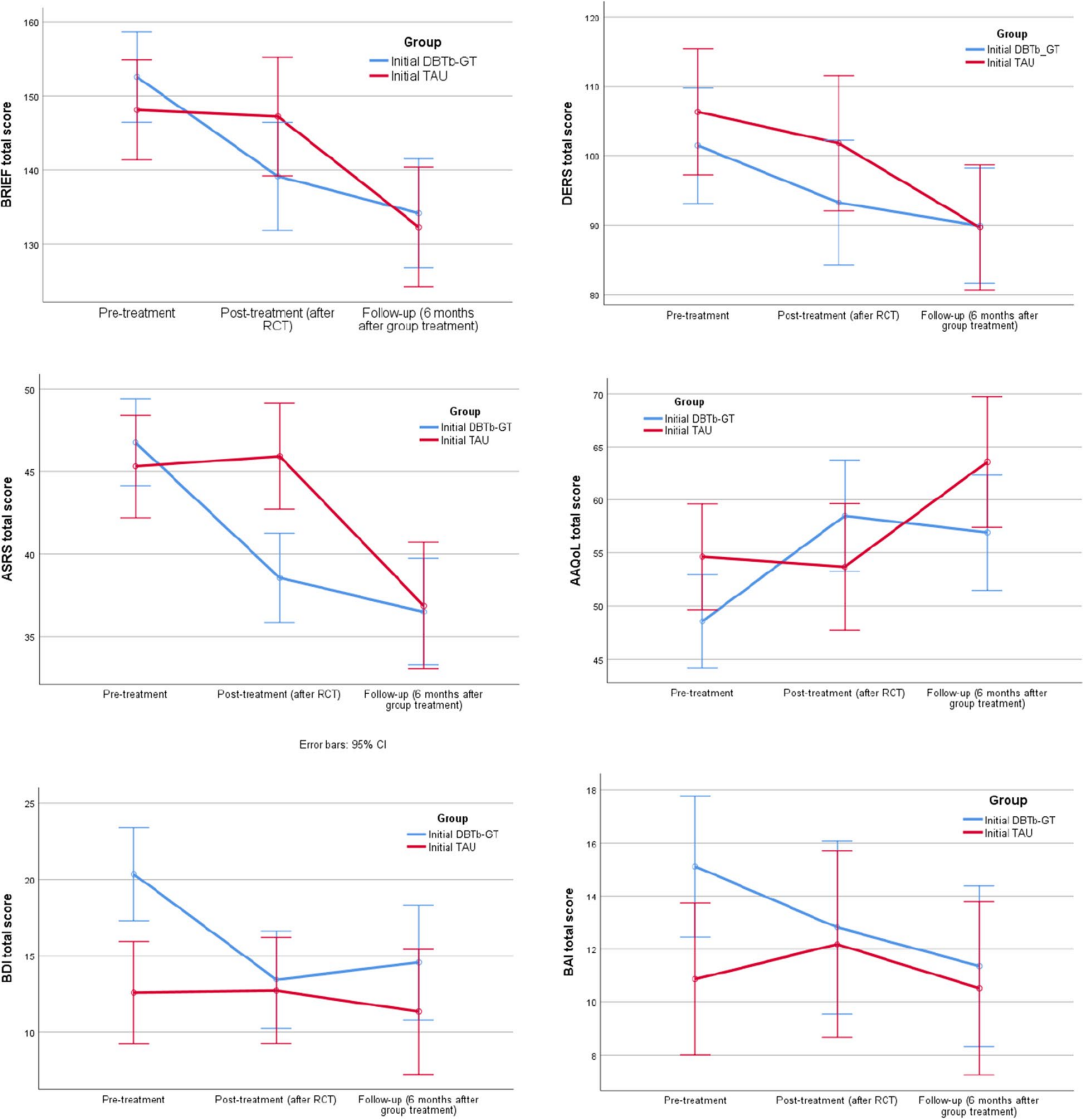
Change in symptom scores from randomized controlled trial and extended follow-up after ended group treatment. The graphs show mean symptom scores for main and secondary outcomes at pre- and post-treatment for the controlled trial (dialectical behavioral therapy based group treatment (DBTb-GT) and Treatment as usual (TAU), and at the extended uncontrolled follow-up, i.e. six months after having received the DBTb-GT for all participants. Analyses and graphs are based on analyses of repeated measures (ANOVA) in SPSS. Bars represent 95% CI. Abbreviations: BRIEF-A = Behavior Rating Inventory of Executive Function-Adult Version, total sum score; DERS = Difficulties in Emotion Regulation Scale, total sum score; ASRS = Adult ADHD Self-Report Scale, total sum score; AAQoL = Adult ADHD Quality of Life Questionnaire, total sum score; BDI = Beck Depression Inventory, total sum score; BAI = Beck Anxiety Inventory, total sum score

Participants receiving TAU in the RCT showed significant and corresponding improvements after completing the post-trial additional 14-week DBT-bGT, and at 6 months follow-up thereafter (Fig. 2).

### Adherence to treatment, feasibility and safety

Among the 121 patients, 10 of the 60 patients (16.7%) randomized to DBT-bGT were registered as ‘drop-outs’, compared to seven of 61 (11.5%) in the TAU group (χ^2^ = 0.68, p = 0.41). Reasons for dropping out of the group treatment were mainly related to practical and psychosocial circumstances, e.g. time schedules at work, sickness, and relational break-ups (see Fig. 1). Only one patient reported the drop-out being related to the treatment (‘too demanding’). The mean number of lost sessions for patients completing the group treatment was 1.38 (range 0–7, median 1), with 85% participating at 12 or more of the 14 sessions. No adverse events related to the DBT-bGT were reported. Five of the seven clinics in the multicenter study have continued offering the group treatment after ended RCT.

According to information from the clinicians following the participants in TAU, the TAU consisted mostly of individual consultations of supportive character, including pharmacological controls and adherence for those using medication. The number of consultations varied from zero (*n* = 1) to weekly (*n* = 3), with a mean of 4.7 and a median of 4 consultations during the 14-week trial period.

Approximately 1 of 3 patients underwent some kind of change in their ADHD-medication during the trial, but the proportions did not differ significantly between the DBT and TAU groups (*n* = 12/29.3% and *n* = 14 /33.3%, respectively, chi-square test *p* = 0.845). Changes included both reductions and increase of dosage, and we could not observe any systematic difference in reported reasons for change in medication between the groups.

## Discussion

This multicenter study is among the largest randomized trials on a psychotherapeutic intervention for adults with ADHD. The main finding was that patients receiving a manualized 14-week DBT-bGT reported significantly better improvements of self-reported executive functioning (EF), core symptoms of ADHD and quality of life compared to patients receiving treatment as usual. Effect sizes of the DBTb-GT were moderate to large. This should be of particular notice, since most of the patients were already stabilized on medication at inclusion. We also found a significant reduction of depressive symptoms. Improvements were maintained six months after ended group treatment in a non-controlled follow-up for all participants after having received DBT-bGT. The change in emotion regulation (ER) according to DERS did not differ between the two treatment groups immediately after treatment, but showed a continued and significant improvement six months after ended group treatment, indicating a possible effect at longer term.

This study is the first to assess primary effects of this specific DBTb-GT on EF and ER among adults with ADHD in a controlled trial. The treatment effect on self-reported EF (according to BRIEF) is thus a novel finding. It is however in line with findings from some studies of related group interventions, like mindfulness-based cognitive therapy [19, 46], and mindfulness meditation training [47], whereas a study on standard CBT did not find any effect on BRIEF [16]. Interestingly, the cited studies showing improvement of EF all included mindfulness as a treatment component, indicating its putative role in ‘brain-training’.

We did not find any significant effect of DBTb-GT on ER. Although in line with a more indirect measure from the COMPAS study (i.e. a subscale of impulsivity and emotional lability) [31], this was somewhat unexpected, since ER is one of the main targets of DBT. Some explanations may be suggested; first, the DBTb-GT for adult ADHD of 14 weeks is of considerably shorter duration than the original DBT for personality disorders, and may thus represent insufficient time or specificity to alleviate emotional problems. Our finding that ER improved at the six months’ follow-up, although the non-controlled nature of this extension prevented us from drawing causal inferences, supports this. The Swedish group found no effect of the DBTb-GT on a Perceived Stress Scale in their controlled study [28], whereas a later uncontrolled study demonstrated significant impact of DBTb-GT on both symptoms of perceived stress, mindful attention and acceptance after 14 weeks [27]. Interestingly, two recent uncontrolled studies of group therapies addressing emotional problems in adults with ADHD showed that 14 weeks may be enough time to improve ER [48, 49].

A second reason for the inconsistencies of effect on ER may be the operationalization of the emotional dysregulation as phenomenon. The DERS questionnaire was not originally developed for adults with ADHD, and may not capture emotional traits most typical for this patient group. We applied DERS because it includes components that are important targets of DBT (e.g. awareness and acceptance of emotions). The two above-mentioned studies targeting ER in adults with ADHD also used DERS: A recent pilot study of group treatment based on a combination of CBT and DBT, found a positive effect on the DERS, which correlated to the amount of mindfulness practiced by the participants during treatment [48]. The other, a larger, multicenter study, examined the effects of the authors’ own developed group therapy (‘Group Therapy for Improving Emotional Acceptance and Regulatory Skills in Adults with ADHD’) based on elements from both CBT, DBT, Acceptance and Commitment Therapy, and Emotion Regulation Group Therapy [49]. They found a significant effect on ER as measured by the DERS. It should be noted that their study included only ADHD patients who had ‘identified problems with emotion regulation difficulties’, and the results may therefore not be directly comparable to ADHD patients in general. Related to this is our finding of a positive effect of DBTb-GT on symptoms of depression. This is in line with the Morgensterns study [27], whereas controlled trials [28, 31, 36] did not find any effect of DBT-based treatment on depressive symptoms in ADHD adults. One explanation may be the lower baseline scores of BDI in the studies with negative findings. Indeed, a mean BDI score of 20 in our treatment groups indicates that some of these patients had scores above the conventional cut-off (i.e. > 20) for a depressive episode. Although these patients were not judged clinically as having a depressive episode that would interfere with treatment, this finding may however motivate future studies to assess the predictive role of depressive symptoms on the effect of this group treatment.

The effect on self-reported ADHD core symptoms was larger in our study than in other controlled trials based on the same treatment manual [28, 31, 50]. The larger effect size found in our study may be due to slight differences in the actual delivered treatment, i.e. 14 sessions instead of 12 and 13 in the COMPAS and Swedish studies, respectively, and, perhaps more importantly, the addition of individual coaching in our study. Another explanation may be the differences in the control conditions, i.e. the non-standardized TAU in our study, versus more standardized general clinical management or discussion groups in the other studies. The COMPAS study did not find any difference in effect between DBTb-GT and general clinical management on clinician-rated core ADHD symptoms [31]. However, the DBTb-GT was more effective on a more general outcome measure, the clinical global impression scale [51]. Further, in one of their follow-up studies assessing the patient’s perspective, the DBTb-GT was rated superior to general clinical management in reducing self-reported ADHD-symptoms, with only small to moderate correlations with the clinician-based measures [52]. One may thus question whether potential benefits of the DBT-bGT may be partly undetected by traditional clinician-based assessment. After all, the explicit goal of this treatment is to learn how to live with and manage symptoms rather than symptom reduction per se [28]. In line with this, a recent feasibility trial of this group treatment found no significant difference on self-reported core symptoms of ADHD, although 88% of the participants reported that they could control their symptoms better after ended group treatment [50].

The finding that participants in the group treatment reported a higher increase of quality of life relative to TAU in our study, is in line with some of the other studies of DBTb-GT [27, 36], but not all. The COMPAS-study found that the increase in quality of life, still significant 1.5 year after ended treatment, was regardless of the initial treatment arm. They argue that the lack of difference between the group-treatment and general clinical management probably reflects a more non-specific treatment effect [53]. Interestingly though, in the context of the earlier discussion on emotion regulation, scores on the quality of life domain specifically related to feelings were more increased among participant that had received the DBT-based group treatment [53].

To learn and practice skills to cope with ADHD symptoms cognitively and emotionally are typically part of several psychosocial treatments based on CBT [54] and, as discussed, ‘third wave’ behavioral interventions based on e.g., meta-cognition, mindfulness and acceptance are increasingly studied [18]. To compare treatments directly head-to-head may however be challenging, due to slight differences in treatment elements and study designs, as well as in the labelling of the intervention. Hence, in this study, different components of the DBT-bGT like the group format, the principle of acceptance, mindfulness exercises, and the individual coaching between group sessions may have beneficial effects on different problems of ADHD. Further studies with specified designs should pursue the question of ‘what works for whom’.

## Limitations and strengths

Evaluating effects of this non-pharmacologic treatment raises methodological issues as related to the complexity of the intervention, the influence of different care providers and expertise of the centers, and the open-label design [55]. Even though we used a manual-based therapy procedure for the DBT-group, and controlled for site in the analyses, sources of variation in the delivering of the treatment may exist. However, our randomized trial design implied a corresponding variation in the comparison group TAU. Because the TAU condition was not a group-therapy setting, we cannot infer specific effects of the DBT-treatment; only superiority of this group-treatment as a whole compared to the individualized TAU.

Another potential limitation of this study is that the TAU-condition was not standardized, and thus could vary from a few to weekly consultations during the 14-week period. Further, since patients in the TAU-group knew they were offered DBT-bGT after the first, randomized phase of the study, some may have perceived TAU more as a ‘waiting list’ condition. This could have lowered their expectancy to the received TAU and potentially influenced their symptom reports after TAU in a negative direction, i.e. in favor of a larger effect size of the DBTb-GT. On the other hand, non-standardized TAU is more representative of clinical reality, making the results relevant for clinical practice.

The main outcome measures in this study were based on self-reported symptoms and functioning. We thus lacked a clinician-based measure, which is generally considered as more objective*.* However, the last decade’s increased focus on patient-centered health-services has led to recommendations of using patient-related outcome measures, particularly when it comes to psychological symptoms [56]. A review of studies on adults with ADHD found an overall good concordance between clinician-based and self-report measures of the same (core) symptoms [57]. On the other hand, the significant reduction in self-reported ADHD-symptoms at follow-up for patients receiving group-treatment in the COMPAS-study was no longer significant when using clinician-rated ADHD-symptoms [51]. The two types of measures probably capture different aspects of the studied phenomenon and may not be directly comparable to each other.

This study has several strengths. It is one of the largest published randomized trials on a psychotherapeutic intervention for adults with ADHD. Further, the multicenter design limits potential therapeutic or clinician-related bias, and the naturalistic setting, i.e. including patients both with and without pharmacological treatment, few exclusion criteria, and clinicians with various professional background and training, increases the generalizability of our results to ordinary clinical settings.

## Conclusions

Overall, this manualized 14-week DBT-based group treatment was effective in improving self-reported executive functioning, core symptoms of ADHD and quality of life in adults with ADHD, with improvements still lasting six months after ended treatment. The lack of effect on emotional regulation immediately after treatment may reflect that emotional problems represent a more complex phenomenon that may require more specific skill training or longer duration. Limitations of the study include the lack of clinician- based outcome measures, lack of standardization of the control condition treatment as usual, and that the six-months follow-up did not include a control condition. Altogether, the design and results of this study indicate that this group treatment is an effective, feasible and well-tolerated non-pharmacological option for adult patients with ADHD.

## Data Availability

The data that supports the findings of this study is prevented from public availability due to the sensitive nature and that participants have not given consent for their data to be shared with other parties. However, upon reasonable request to the corresponding author, the local Data Protection Officer will consider data availability subject to approval. The full trial protocol is available from the corresponding author on request.

## Abbreviations

ADHD: Attention Deficit Hyperactivity Disorder
ANOVA: Analyses of variance
ASRS: Adult ADHD Rating Scale
AAQoL: Adult ADHD Quality of Life Scale
AUDIT: Alcohol Use Disorder Identification Test
BAI: Becks Anxiety Inventory
BDI: Becks Depression Inventory
BRIEF-A: Behavior Rating Inventory of Executive Function, adult version
CBT: Cognitive behavioral therapy
CI: Confidence interval
COMPAS-study: Comparison of Methylphenidate and Psychotherapy in Adult ADHD Study
DBT: Dialectical Behavioral Therapy
DBTb-GT: Dialectical Behavioral Therapy-based group treatment
DERS: Difficulties in Emotion Regulation Scale
DUDIT: Drug Use Identification Test
EF: Executive functioning
ER: Emotion regulation
ES: Effect Size
SD: Standard deviation
TAU: Treatment as usual
